# Improved vision transformer for sports image classification via transfer learning and knowledge distillation

**DOI:** 10.1371/journal.pone.0337744

**Published:** 2026-10-16

**Authors:** Chao Li, Daniel Memmert, Qiang Liu

**Affiliations:** 1 School of Physical Education, Jining University, Qufu, China; 2 Faculty of Educational Sciences and Technology, Universiti Teknologi Malaysia, Skudai, Malaysia; 3 Institute of Exercise Training and Sport Informatics, German Sport University Cologne, Cologne, Germany; 4 School of Physical Education, Shandong University, Jinan, China; Centro de Investigacion en Ciencias de Informacion Geoespacial AC (Research Center on Geospatial Information Sciences), MEXICO

## Abstract

To address the challenges posed by complex visual characteristics in sports image classification, this study proposes an enhanced Vision Transformer (ViT) model built upon the Native Resolution ViT (NaViT) architecture. The principal innovations include replacing conventional single-layer feedforward networks with parallel feedforward networks and introducing an attention-guided distillation framework with input-dependent attention-weighted feature fusion, which adaptively regulates the contribution of frozen distillation features to the prediction pathway. The proposed approach leverages ImageNet-pretrained weights and performs domain-specific fine-tuning on sports datasets. Experimental results demonstrate substantial performance gains. On the multi-sport classification dataset, the proposed model achieves an accuracy of 0.9752 and a Macro-F1 score of 0.9753, outperforming the evaluated CNN benchmarks and ViT variants. For soccer match event classification, it achieves an accuracy of 0.9863 and a Macro-F1 score of 0.9718, while ranking first in the TOPSIS evaluation. These results demonstrate the effectiveness of integrating parallel feedforward processing, transfer learning, and distillation-guided feature fusion for sports image classification under complex visual conditions.

## 1 Introduction

In recent years, with the continuous development of computer vision technologies, image classification has become a key research area in artificial intelligence [1]. The sports domain has seen a significant increase in video and image data, offering substantial resources for applications like motion analysis [2], event monitoring [3], and action recognition [4]. As a specialized subfield of computer vision, sports image classification enables real-time monitoring and intelligent analysis in competitive environments, while also proving valuable for athletic performance evaluation, training assessment, and athlete behavior recognition [5]. However, the inherent complexity of sports images—marked by dynamic content variations, cluttered backgrounds, diverse athlete movements, and frequent posture changes—poses considerable challenges for accurate and efficient classification.

While traditional Convolutional Neural Networks (CNNs) have achieved notable successes in image classification [6,7], their limitations in capturing global features and long-range dependencies, particularly in maintaining detailed contextual information, have become apparent. ViT present an innovative architectural approach that addresses these limitations through self-attention mechanisms [8]. By dividing images into fixed-size patches and processing them with linear embeddings and positional encoding as Transformer inputs, ViT effectively models both local features and global dependencies [9]. Despite showing improved performance over conventional CNNs in various vision tasks, applying standard ViT models to sports image classification faces three main challenges: 1) difficulty in distinguishing sports categories and event types that share similar visual cues; 2) A single ViT architecture may lead to missed and false detections when handling complex sports images with diverse postures; and 3) sensitivity to environmental disturbances such as illumination variations (affecting pixel intensity distributions) and occlusions (leading to partial feature loss or key region masking).

To address these issues, this study proposes an improved ViT architecture combining transfer learning and knowledge distillation for sports image classification. We first utilize a ViT model pre-trained on large-scale datasets, adapting its general image feature extraction capabilities to sports classification through transfer learning, thereby reducing reliance on extensive domain-specific annotations. Then, we implement an encoder-based knowledge distillation method that transfers temperature-softened supervisory information from a fixed teacher model to the student model during training; in the experiments, the corresponding distillation-oriented variant achieved better performance than the baseline NaViT. The combined approach offers an effective optimization strategy.

The main contributions of this work include:

(1) An improved network architecture building on NaViT improvements, replacing single-layer feedforward networks with parallel feedforward networks combined with multi-head attention structures, alongside optimized encoding configurations for better knowledge distillation compatibility.
(2) An encoder-based knowledge distillation framework integrates input-dependent, attention-weighted feature fusion with customized loss functions, adaptively modulating the contribution of frozen distillation features to the prediction pathway during fine-tuning.
(3) A refined training approach integrating ImageNet pre-trained model fine-tuning with the proposed distillation method, achieving competitive accuracy and robustness in sports image classification.
(4) Comprehensive validation across multiple public datasets and real-world collected data, demonstrating the model’s effectiveness in complex sports scenarios and practical deployment potential.

This research provides an effective solution for sports image classification while contributing insights for adapting ViT architectures to specialized domains. Future directions may involve integrating multimodal learning and further architectural refinements for broader sports applications.

The remainder of this paper is organized as follows: Section 2 reviews related studies on sports image classification, CNN-based models, ViT architectures, NaViT, transfer learning, and knowledge distillation. Section 3 describes the proposed method, including hierarchical image preprocessing, the improved NaViT-based network architecture, transfer learning strategy, and encoder-based knowledge distillation framework. Section 4 presents the experimental settings, evaluation metrics, comparative results, and discussion on both general sports event classification and soccer match event classification. Section 5 discusses the mechanisms underlying the observed performance and the limitations of the proposed framework. Finally, Section 6 summarizes the main findings, contributions, and future research directions.

## 2. Related work

Sports image classification is a specialized image classification task that assigns a semantic label to a still image depicting a sports scene, sport category, athlete action, or match event. It differs from general-purpose image classification because sports images often contain highly dynamic body movements, similar playing fields, cluttered backgrounds, motion blur, occlusion, viewpoint changes, and large intra-class variations. It should also be distinguished from video-based action recognition: sports image classification focuses on recognizing semantic categories from individual images, whereas video-based recognition relies on temporal information across successive frames. Owing to its value in sports media indexing, event retrieval, athlete-performance analysis, and intelligent sports-content management, sports image classification has become an important topic in computer vision research [5,10–13].

Early studies on sports visual recognition mainly relied on handcrafted features such as color, texture, edges, object shape, and scene composition. These methods are interpretable and computationally simple, but their performance depends heavily on feature design and is often limited when different sports share similar visual appearances. For example, sports such as soccer, rugby, American football, and field hockey may contain similar grass fields, player distributions, and motion patterns, making them difficult to distinguish using shallow features alone. With the development of deep learning, CNN-based models have become dominant because they can learn hierarchical visual representations directly from image data.

Several studies have explored CNN-based sports image classification. Joshi et al. [10] used InceptionV3 as a feature extractor and neural networks as classifiers for six sports categories, showing that deep CNN features outperform traditional classifiers such as Random Forest, KNN, and SVM. Podgorelec et al. [11] proposed a CNN transfer-learning framework with differential-evolution hyperparameter optimization for classifying visually similar sports disciplines. Zhou et al. [12] combined deep neural networks with a novel tuna swarm optimization algorithm to improve sports image classification performance. More recently, Li et al. [14] introduced an SE-RES-CNN model that integrates channel attention and residual learning for large-category sports image classification. These studies demonstrate that CNNs are effective for extracting local and hierarchical features from sports images, but they may still be limited in modeling long-range dependencies and global semantic relationships.

Specially, CNNs remain sensitive to diverse image transformations such as rotation, scaling, and illumination variations [15]. In sports imagery, factors like changing field conditions, player orientations, and lighting can lead to feature degradation, adversely impacting classification accuracy. To address partial feature loss, Meng et al. [16] proposed a dual-fusion architecture combining 1×1 convolutions with hyperconnections to strengthen feature representation. Additionally, CNNs exhibit limitations in high-level semantic understanding, such as contextual interpretation of actions (e.g., goals, fouls, assists) in sports scenarios. Raabe et al. [17] tackled this challenge by developing a graph-structured CNN variant to recognize and annotate diverse behaviors in soccer matches.

To overcome the limitations of CNNs, self-attention-based architectures have been introduced into visual recognition tasks. ViT treats an image as a sequence of patches and uses self-attention to model relationships among distant regions [18]. This mechanism is useful for sports images because discriminative cues may appear in spatially separated regions, such as the athlete’s posture, sports equipment, field layout, and background context. NaViT further improves the flexibility of ViT by supporting native-resolution and variable-aspect ratio inputs, making it suitable for images collected from different sources and resolutions [19]. Other ViT variants, such as DeiT and deeper ViT architectures, have shown that data-efficient training and architectural refinement can improve Transformer-based image recognition [20,21].

Knowledge distillation is another important strategy for improving model training and deployment efficiency. In the student-teacher paradigm, a larger or better-trained teacher model provides soft supervisory signals to guide a smaller or task-specific student model. The teacher’s softened output distribution contains inter-class similarity information, which can help the student learn richer decision boundaries than those provided by one-hot labels alone [22]. This idea is especially relevant to sports image classification, where visually similar sports or events may have subtle differences in posture, context, or object interactions.

In summary, existing sports image classification studies have made progress through CNN feature extraction, transfer learning, attention mechanisms, and optimization-based training. However, many methods still focus mainly on local convolutional features or require extensive hyperparameter search, while fewer studies jointly address multi-resolution sports images, global contextual modeling, and efficient teacher-guided learning. To address these limitations, this study proposes a knowledge-distillation-improved NaViT framework that combines transfer learning, native-resolution image processing, self-attention-based global feature modeling, and encoder-based student-teacher supervision.

## 3. Methods and materials

This study proposes an improved ViT framework integrating transfer learning and knowledge distillation, designed to systematically harness knowledge from pre-trained and teacher models for improved recognition of sports images across diverse resolutions. The methodology comprises four core stages: 1) Application of standardized preprocessing to input images; 2) Construction of an optimized network architecture based on NaViT; 3) Transfer learning via a dual-head ViT—trainable prediction head plus frozen benchmark head aligned through a learnable mapping; 4) Encoding-based knowledge distillation by concatenating teacher embedding with class and patch tokens and training dual heads under softened supervision.

The complete framework is illustrated in Fig 1.

**Fig 1:**
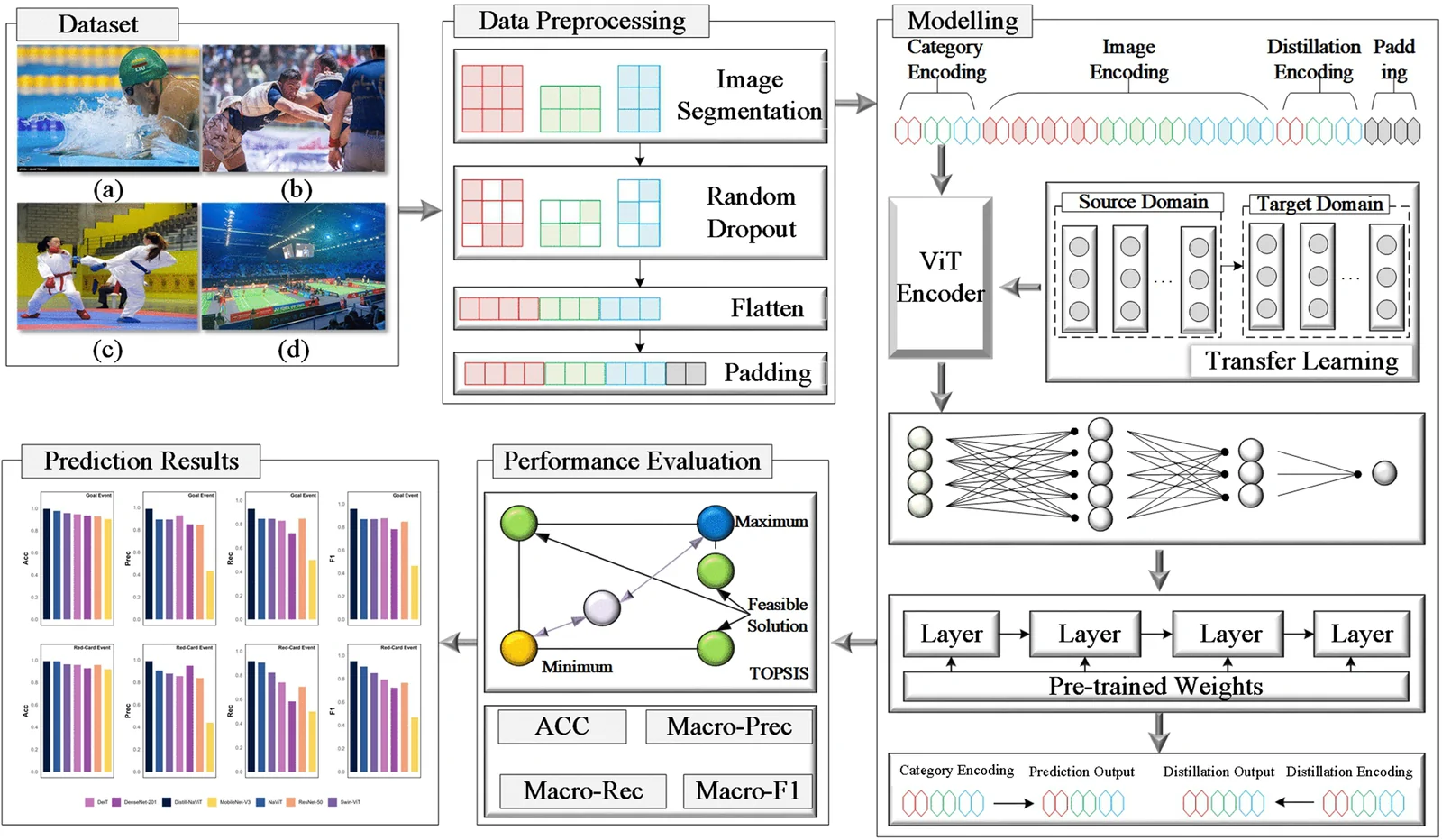
The framework of the proposed method. (The composite layout, annotations, and graphical elements were created by the authors. The sport photographs in panels (a)–(d) were adapted from Wikimedia Commons under the Creative Commons Attribution 4.0 International (CC BY 4.0) License: (a) Javid Nikpour; (b) Ali Dalkabadi; (c) Wolfgang Burghardt; and (d) Hoolen. The photographs were cropped/resized and arranged in the composite figure where applicable. Full source URLs and license information are provided in the Image Attributions section.).

### 3.1. Image preprocessing with hierarchical encoding

During the data preprocessing phase, input sports images with varying resolutions are mathematically represented as a tensor: $X\in {\mathbb{R}}^{H\times W\times C},$ where *H*, *W*, and *C* respectively denote the image height, width, and color channels. To enable multi-resolution compatibility, we adopt a patch-based processing strategy. Following the empirical findings of Dosovitskiy et al., each image is systematically divided into non-overlapping 16×16 pixel patches. This specific patch size has been experimentally validated to balance computational efficiency and feature representation capability. Let $N=\frac{H}{16}\times \frac{W}{16}$ represent the total number of patches, where each patch is defined as: $x_{i}\in {\mathbb{R}}^{16\times 16\times C},\;i=1,2,...,N.$

Each patch is then flattened into a 1D vector $x_{i}^{\prime }\in {\mathbb{R}}^{256C}$ and linearly transformed into a lower-dimensional latent space through an embedding layer: $z_{i}=W_{p}\;x_{i}^{\prime }+b_{p},$ where $W_{p}\in {\mathbb{R}}^{D\times 256C}$ is the projection matrix, $b_{p}\in {\mathbb{R}}^{D}$ the bias vector, and *D* the embedding dimension [23]. This projection reduces dimensionality while preserving critical visual patterns.

To improve model robustness, we implement a stochastic patch dropout mechanism during training. Randomly selected patches are replaced with a learnable null embedding $e_{\text{null}}\in {\mathbb{R}}^{D}$, simulating occlusions and encouraging the model to focus on discriminative regions. Spatial relationships between patches are preserved through additive positional encodings $E_{i}\in {\mathbb{R}}^{D}$. Since self-attention is permutation-invariant and does not inherently encode the order or location of input tokens, each patch embedding $z_{i}$ is added to a position-specific encoding $E_{i}$ according to its original location in the image grid:


$$\begin{array}{c} {\hat{z}}_{i}=z_{i}+E_{i}. \end{array}$$

In this way, the resulting token representation contains both visual content and spatial location information, enabling the Transformer to learn geometric relationships among patches during attention computation. These encodings enable the model to understand geometric arrangements despite the permutation-invariant nature of Transformer architectures.

The complete input sequence integrates two special tokens for improved learning: a class token ($e_{\text{cls}}\in {\mathbb{R}}^{D}$) prepended to capture global semantic information, and a distillation token ($e_{\text{teacher}}\in {\mathbb{R}}^{D}$) appended to receive knowledge from the teacher model via attention mechanisms.

The final encoded sequence is structured as:


$$\begin{array}{c} Z=[e_{\text{cls}},\;{\tilde{z}}_{1},\;{\tilde{z}}_{2},\;...,\;{\tilde{z}}_{N},\;e_{\text{teacher}},\;\{e_{\text{null}}\}], \end{array}$$

where ${\tilde{z}}_{i}$ denotes patches after dropout processing. This hierarchical encoding scheme simultaneously preserves local visual features (${\tilde{z}}_{i}$), global semantics (*e*_cls_), transferred knowledge (*e*_teacher_), and robustness patterns (*e*_null_).

### 3.2. Improved network architecture based on NaViT

The improved architecture builds upon the NaViT framework while preserving the fundamental ViT structure, consisting of multiple stacked sub-ViT layers. Each sub-layer integrates a multi-head self-attention (MHSA) encoder followed by two parallel feedforward blocks.

For the input encoding sequence $Z\in {\mathbb{R}}^{L\times D}$, where *L* denotes the sequence length and *D* denotes the embedding dimension, layer normalization is first applied. The MHSA mechanism generates the query *Q*, key *K*, and value *V* representations through linear projections:


$$\begin{array}{c} Q=ZW_{Q},\;K=ZW_{K},\;V=ZW_{V}, \end{array}$$

where $W_{Q},W_{K},W_{V}\in {\mathbb{R}}^{D\times D}$ are trainable projection matrices. Thus, $Q,K,V\in {\mathbb{R}}^{L\times D}$. These representations are then divided into *h* parallel attention heads, with each head having dimension *d* = *D*/*h*. For the *i*-th head:


$$\begin{array}{c} Q_{i}=Q[:,id:(i+1)d],\;K_{i}=K[:,id:(i+1)d],\;V_{i}=V[:,id:(i+1)d], \end{array}$$

where $Q_{i},K_{i},V_{i}\in {\mathbb{R}}^{L\times d}$.

Each head computes scaled dot-product attention:


$$\begin{array}{c} \text{Attention}(Q_{i},K_{i},V_{i})=\text{softmax}\left(\frac{Q_{i}K_{i}^{T}}{\sqrt{d}}\right)V_{i}. \end{array}$$

The outputs from all heads are concatenated and linearly transformed: $\text{MHSA}(Z)=\text{Concat}({\text{Head}}_{1},...,{\text{Head}}_{h})W_{O},$ where $W_{O}\in {\mathbb{R}}^{h\cdot d\times D}$ is the output projection matrix.

After multi-head self-attention, a residual connection and layer normalization are applied:


$$\begin{array}{c} H_{attn}=LayerNorm\left(MHSA(Z)+Z\right). \end{array}$$

The attention-enhanced representation is then routed to two parallel feedforward blocks: a trainable prediction block and a frozen distillation block. The two blocks use independent parameter sets and do not share weights. Specifically, both blocks receive the attention-enhanced representation $H_{attn}$ as input, such that


$$\begin{array}{c} H_{pred}^{\left(0\right)}=H_{dist}^{\left(0\right)}=H_{attn}. \end{array}$$

The outputs of the two feedforward blocks are then defined as


$$\begin{array}{c} H_{pred}=F_{pred}\left(H_{pred}^{\left(0\right)};\theta_{pred}\right),\;H_{dist}=F_{dist}\left(H_{dist}^{\left(0\right)};{\bar{\theta }}_{dist}\right). \end{array}$$

For b∈{pred,dist}, each feedforward block is defined as


$$\begin{array}{c} F_{b}(H;\theta_{b})=LayerNorm\left[ReLU\left(LayerNorm(H)W_{b,1}+b_{b,1}\right)W_{b,2}+b_{b,2}+H\right], \end{array}$$

where $W_{b,1}\in {\mathbb{R}}^{D\times 4D}$ and $W_{b,2}\in {\mathbb{R}}^{4D\times D}$. The prediction- and distillation-block parameters are independent. Within the two parallel feedforward blocks, only the prediction-block parameters are updated during fine-tuning, whereas the distillation-block parameters remain frozen:


$$\begin{array}{c} \theta_{pred}^{\left(t+1\right)}=\theta_{pred}^{\left(t\right)}-\eta \nabla_{\theta_{pred}}{ℒ}_{total},\;{\bar{\theta }}_{dist}^{\left(t+1\right)}={\bar{\theta }}_{dist}^{\left(t\right)}. \end{array}$$

Attention-weighted feature fusion transfers information from the frozen distillation block to the trainable prediction block. The prediction representation is used as the query, while the frozen distillation representation provides the key and value:


$$\begin{array}{c} Q_{pred}=H_{pred}W_{Q}^{f},\;K_{dist}=\mathrm{sg}(H_{dist})W_{K}^{f},\;V_{dist}=\mathrm{sg}(H_{dist})W_{V}^{f}, \end{array}$$

where sg(·) denotes the stop-gradient operation, $d_{f}$ denotes the feature dimension used in the fusion attention, $W_{Q}^{f},W_{K}^{f},W_{V}^{f}\in {\mathbb{R}}^{D\times d_{f}}$, and $W_{O}^{f}\in {\mathbb{R}}^{d_{f}\times D}$ denotes the corresponding output projection matrix. The attention weights and transferred distillation features are computed as


$$\begin{array}{c} A_{dist\to pred}=softmax\left(\frac{Q_{pred}K_{dist}^{T}}{\sqrt{d_{f}}}\right), \end{array}$$

$$\begin{array}{c} H_{transfer}=A_{dist\to pred}V_{dist}W_{O}^{f}. \end{array}$$

The transferred features are fused with the prediction representation through a residual connection:


$$\begin{array}{c} {\tilde{H}}_{pred}=LayerNorm\left(H_{pred}+H_{transfer}\right), \end{array}$$

and the prediction output is obtained as


$$\begin{array}{c} y_{pred}=g_{pred}\left(Pool\left({\tilde{H}}_{pred}\right)\right), \end{array}$$

where Pool(·) denotes the pooling operation and $g_{pred}(\cdot )$ denotes the prediction head.

Because $A_{dist\to pred}$ is recomputed for each input, the contribution of the frozen distillation features is adjusted dynamically according to the current prediction representation. This architecture is illustrated in Fig 2.

**Fig 2:**
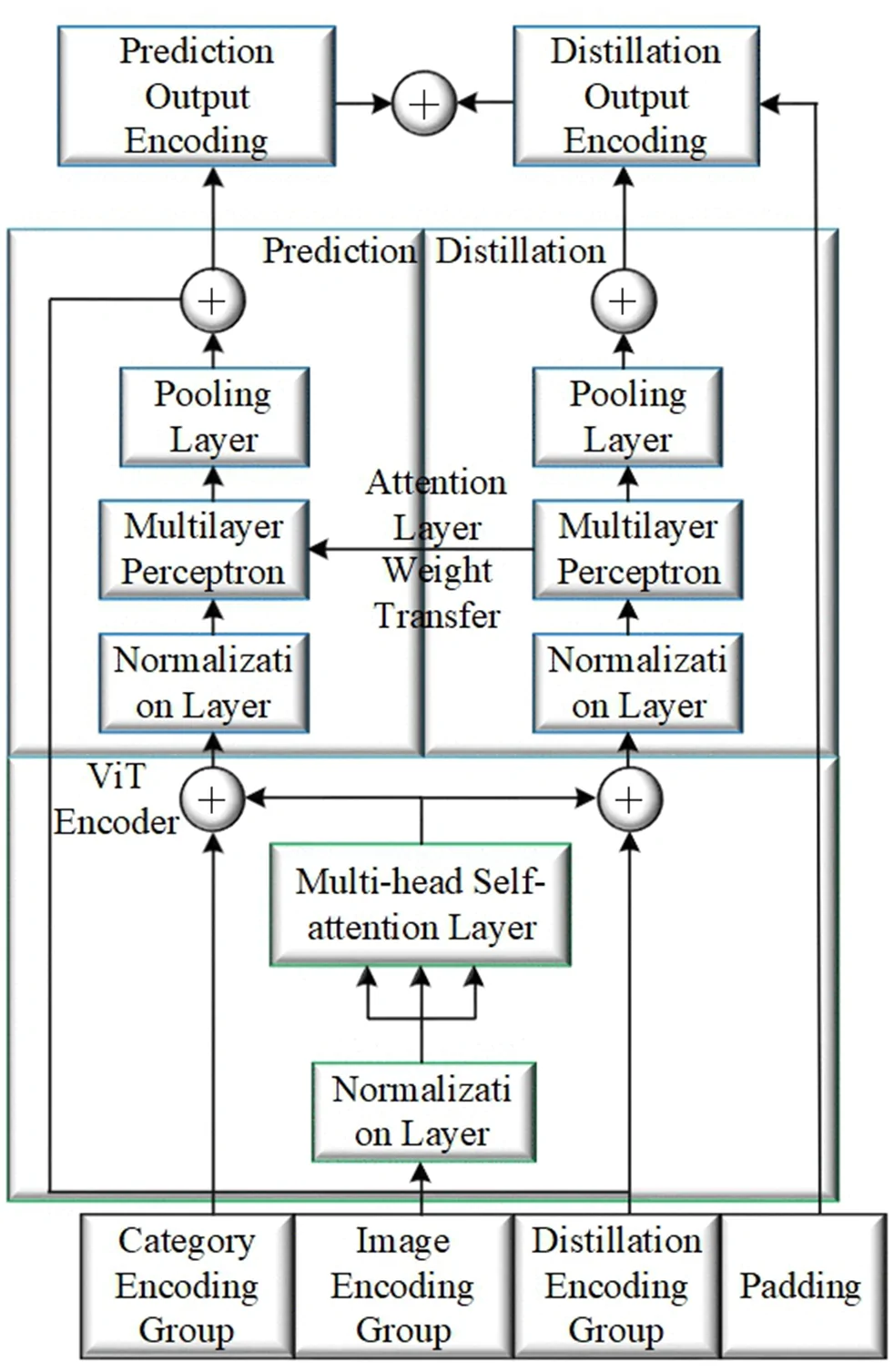
The architecture of the improved NaViT network.

### 3.3. Transfer learning

To further accelerate model convergence and improve overall performance, this paper employs a ViT model pre-trained on ImageNet for transfer learning. First, the pre-trained ViT extracts both intermediate features and prediction outputs from the input samples. We use a special softmax-based dual-head classifier consisting of a prediction head and a benchmark (frozen) head. The prediction head yields the model’s actual output, denoted $y_{pred}$, whereas the benchmark head remains frozen during fine-tuning.

To align the student’s prediction logits with the teacher-logit space, we introduce a transfer matrix $W_{trans}$ and bias $b_{trans}$, and define the mapped student prediction as


$$\begin{array}{c} y_{mapped}=W_{trans}\;y_{pred}+b_{trans}. \end{array}$$

We then define a transfer loss to minimize the Euclidean distance between the mapped student prediction and the DenseNet-201 teacher logits:


$$\begin{array}{c} Loss_{transfer}={\left\|y_{teacher}-y_{mapped}\right\|}_{2}^{2}, \end{array}$$

where $y_{teacher}$ denotes the logits produced by the fixed DenseNet-201 teacher model.

The transfer learning procedure follows the fine-tuning process described below. First, the ImageNet-pretrained ViT is loaded as the initialization of the student network, and the original classification layer is replaced with the proposed dual-head classifier, with the number of output units set to match the target sport categories. During training, each mini-batch of preprocessed images is forwarded through the pretrained backbone and the dual-head classifier. The prediction head parameters, the transfer mapping parameters $W_{trans}$ and $b_{trans}$, and all trainable parameters of the student backbone are updated via backpropagation. The benchmark head remains frozen throughout training, while the transfer loss aligns the mapped student prediction with the fixed DenseNet-201 teacher logits.

Fig 3 illustrates the architecture of the transfer learning framework and the relationships among its principal components.

**Fig 3:**
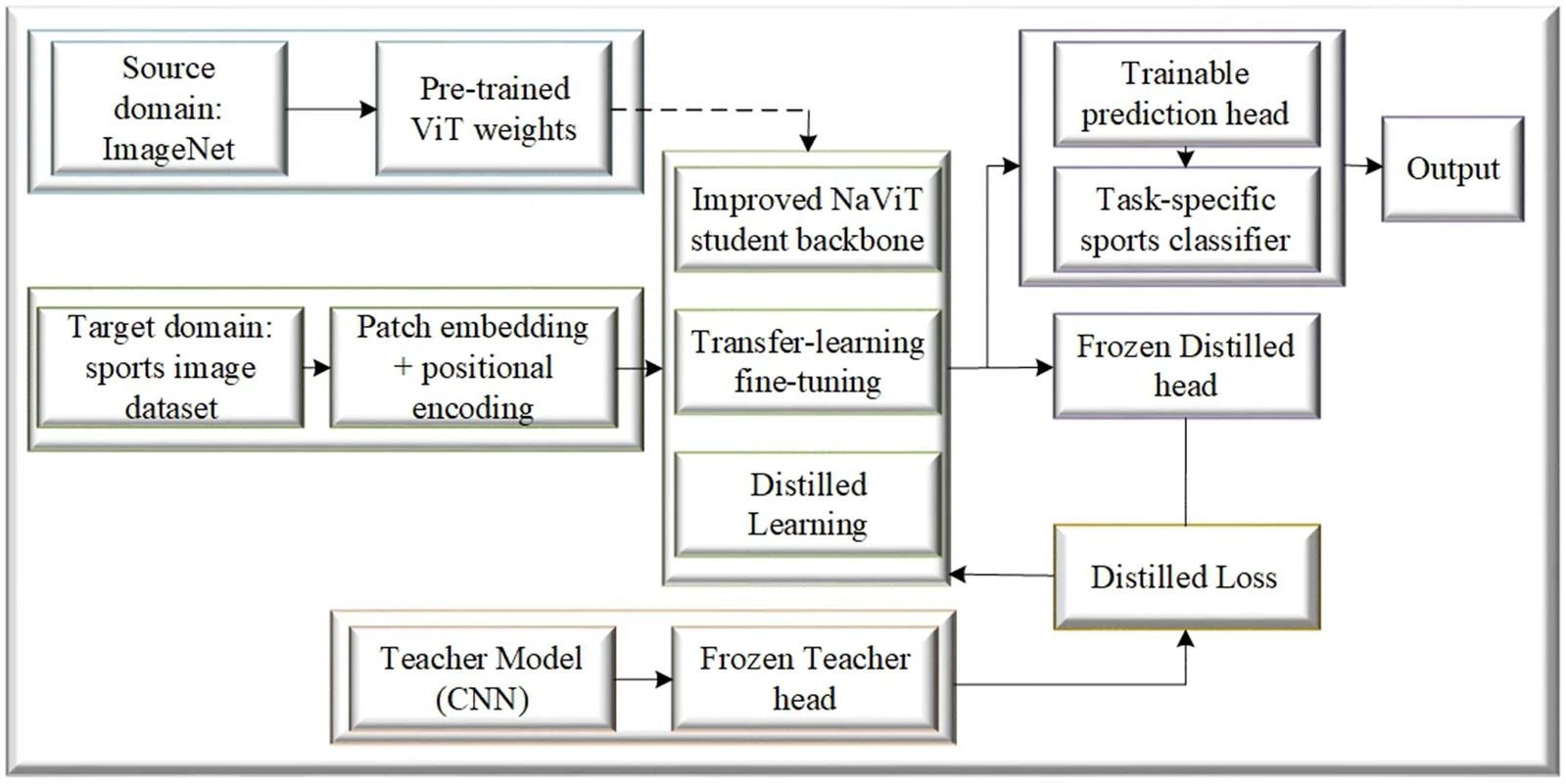
The architecture of the transfer learning framework.

### 3.4. Encoding-based knowledge distillation

Our encoding-based knowledge distillation uses a pre-trained teacher model’s predictions as auxiliary supervision for the student. Hinton et al. [24] mentioned that a well-trained teacher model provides not only the hard class label, but also a softened output distribution that contains inter-class similarity information. Compared with one-hot labels, the teacher’s soft probability distribution provides richer supervisory information by encoding the teacher’s relative confidence across both target and non-target classes, including visually similar categories. This property is particularly useful for sports image classification, where different sports or events may share similar backgrounds, body postures, motion patterns, and visual contexts.

In this study, DenseNet-201 is used as the teacher model because it is a mature CNN architecture with strong hierarchical feature extraction ability and stable classification performance in the comparative experiments. The improved NaViT model is used as the student model. During the distillation stage, the teacher model is kept fixed and only provides supervisory logits, whereas the student model is optimized by backpropagation. The teacher is used only during training and is not part of the student inference path.

For each input image *X*, the fixed DenseNet-201 teacher first performs a forward pass and produces supervisory logits $y_{teacher}$:


$$\begin{array}{c} y_{teacher}=f_{teacher}(X). \end{array}$$

These logits are not used as the final prediction of the proposed model. Instead, they serve as soft supervisory information that reflects the teacher model’s confidence distribution over all candidate classes. Compared with a one-hot class label, $y_{teacher}$ contains richer inter-class similarity information, which helps the student model learn relationships among visually similar sports categories or events.

To make the teacher information compatible with the token-based input format of the ViT student, the teacher logits are projected into the same embedding space as the student tokens:


$$\begin{array}{c} e_{teacher}=\phi_{teacher}(y_{teacher}), \end{array}$$

where $\phi_{teacher}(\cdot )$ denotes the encoding or projection operation, and $e_{teacher}$ is the distilled teacher representation. This representation acts as a distillation token that carries the teacher’s category-level knowledge into the student network.

The distilled teacher representation is then concatenated with the preprocessed class token $e_{true}$ and the image patch encodings ${\tilde{z}}_{i}$ to construct a unified token sequence:


$$\begin{array}{c} Z_{distill}=[e_{true},\;{\tilde{z}}_{1},\;{\tilde{z}}_{2},\;...,\;{\tilde{z}}_{N},\;e_{teacher},\;\{e_{null}\}]. \end{array}$$

In this sequence, $e_{true}$ provides the class-level supervisory token, ${\tilde{z}}_{i}$ represents the encoded visual patches after preprocessing, $e_{teacher}$ introduces the teacher’s distilled knowledge, and $e_{null}$ denotes null or padded positions introduced by patch dropout or variable-resolution processing.

Because some token positions may correspond to blank, padded, or irrelevant patches, a mask is applied before the student encoder processes the sequence. This mask prevents invalid tokens from participating in the attention calculation, thereby reducing noise from non-informative regions and ensuring that the student focuses on valid visual patches and the teacher-provided distillation token.

The teacher logits are converted into a softened probability distribution using temperature-scaled softmax:


$$\begin{array}{c} q_{teacher,j}=\frac{\exp(y_{teacher,j}/T)}{\sum_{k}\exp(y_{teacher,k}/T)}, \end{array}$$

where *T* is a temperature parameter. A larger *T* produces a smoother probability distribution and makes the relative similarities among non-target classes more visible to the student model.

The masked sequence is fed into a ViT-based student initialized with pre-trained weights, which produces two outputs:

(1) A main classification output $y_{pred}$, trained via cross-entropy against the ground-truth label $y_{true}$:

$$\begin{array}{c} Loss_{\text{pred}}=-\sum_{i=1}^{n}y_{true}^{\left(i\right)}\log\left(y_{pred}^{\left(i\right)}\right). \end{array}$$

(2) A distillation output $y_{distill}$, whose corresponding probability distribution is

$$\begin{array}{c} q_{distill,j}=\frac{\exp(y_{distill,j}/T)}{\sum_{k}\exp(y_{distill,k}/T)}. \end{array}$$

The distillation loss is then defined using the softened teacher distribution:


$$\begin{array}{c} Loss_{\text{distill}}=-\sum_{j}q_{teacher,j}\log q_{distill,j}. \end{array}$$

We combine these losses as


$$\begin{array}{c} Loss_{\text{main}}=W\;Loss_{\text{pred}}+(1-W)\;Loss_{\text{distill}}, \end{array}$$

where *W* weights the classification loss and (1−W) weights the distillation loss. To further align the teacher and student prediction distributions, the softened student distribution is computed directly from the prediction logits $y_{pred}$:


$$\begin{array}{c} q_{student,j}=\frac{\exp(y_{pred,j}/T)}{\sum_{k}\exp(y_{pred,k}/T)}. \end{array}$$

We then quantify the divergence between the teacher and student distributions using the Kullback–Leibler (KL) divergence and incorporate it, together with the transfer loss defined in Section 3.3, into the total objective:


$$\begin{array}{c} Loss_{\text{total}}=Loss_{\text{main}}+\lambda_{1}Loss_{\text{transfer}}+\lambda_{2}T^{2}\sum_{j}q_{teacher,j}\log\frac{q_{teacher,j}}{q_{student,j}}. \end{array}$$

Here, $\lambda_{1}$ and $\lambda_{2}$ balance the transfer and distillation terms, respectively, while the factor *T*^2^ compensates for the gradient scaling introduced by temperature.

We optimize the entire training procedure with the AdamW optimizer and incorporate an input-dependent attention-weighted feature fusion mechanism to regulate the contribution of the frozen distillation features to the prediction pathway. The architecture of the encoding-based knowledge distillation approach is illustrated in Figure 4.

**Fig 4:**
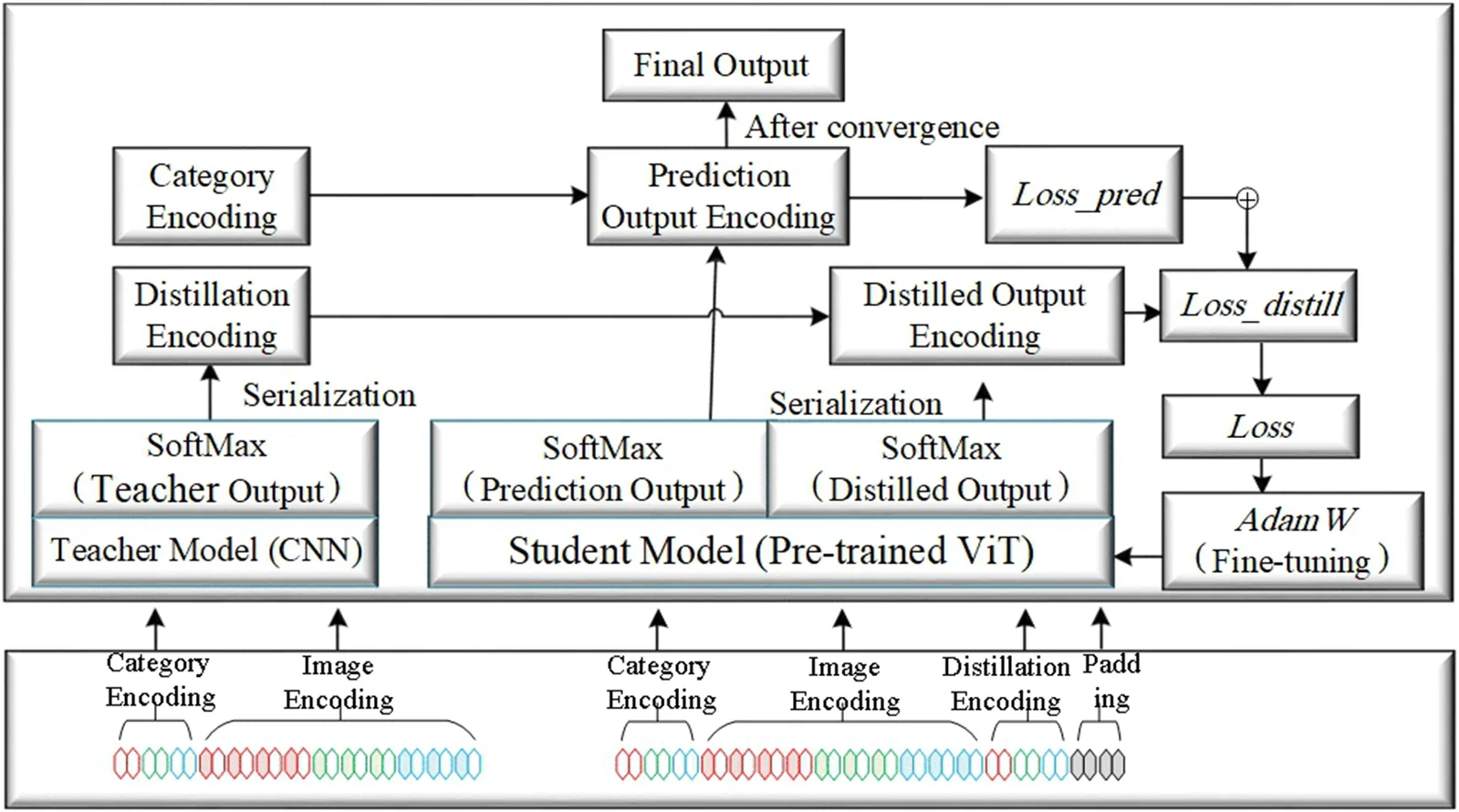
The architecture of the encoding-based knowledge distillation approach.

## 4. Experiments and results

This section evaluates the proposed improved ViT model through a series of controlled experiments. We first describe the experimental environment and unified training parameters to facilitate reproducibility and fair comparison across models. We then introduce the evaluation metrics used to assess classification performance from both individual and composite perspectives. Finally, two experimental scenarios are presented: general sports event classification across multiple sports categories and fine-grained soccer match event classification. These experiments are designed to examine not only the overall accuracy of the proposed model, but also its robustness under practical sports-image conditions involving posture changes, viewpoint differences, background interference, and event-level visual ambiguity.

### 4.1. Experimental environment and parameters

The experimental hardware configuration comprised an Intel® Core™ i7-13700F CPU (2.10 GHz), NVIDIA GeForce RTX 4080 GPU (16GB VRAM), and 32GB DDR4 RAM, operating on Windows 10 Pro 22H2. The software environment utilized Python 3.11.5 with PyTorch 2.0.1 (CUDA 11.8 backend) for accelerated computation. To ensure the scientific rigor and comparability of the experimental results, a uniform set of hyperparameters was applied across all models. The specific settings are detailed in Table 1 below.

**Table 1:** Experimental hyperparameter settings.

Hyperparameter	Value	Remarks
Learning rate	1.00E-05	
Optimizer	AdamW	
Pooling layer	Global average pooling	
Normalization layer	Batch normalization	
Number of ViT layers	5	Not for CNNs (e.g., ResNet)
Feedforward network	Multi-Layer perceptron	
Number of training epochs	100	
Batch size	32	

### 4.2. Performance evaluation

To evaluate the model’s effectiveness, we employ four conventional classification metrics, accuracy, macro-precision, macro-recall, and macro-F1, alongside a composite score that serves as an additional, fifth evaluation metric. This composite score is derived from the entropy-weighted Technique for Order Preference by Similarity to Ideal Solution (TOPSIS) method [25]. Conventional metrics may not always yield consistent model rankings, particularly in multi-class sports image classification, where category imbalance, visually similar classes, and event-level ambiguity can affect precision and recall in different ways. Therefore, TOPSIS is introduced as a complementary, comprehensive evaluation tool rather than as a replacement for the four conventional metrics.

Accuracy measures the overall correctness of classification results. Macro-Precision quantifies the average proportion of truly positive predictions among all positive predictions across classes. Macro-Recall measures the model’s average ability to correctly identify actual positive samples per class. Macro-F1-score is the harmonic mean of Macro-Precision and Macro-Recall, balancing both aspects. They are defined as follows:


$$\begin{array}{c} Acc=\frac{TP+TN}{TP+TN+FP+FN}, \end{array}$$

$$\begin{array}{c} \text{Macro-Prec}=\frac{1}{C}\sum_{i=1}^{C}\frac{TP_{i}}{TP_{i}+FP_{i}}, \end{array}$$

$$\begin{array}{c} \text{Macro-Rec}=\frac{1}{C}\sum_{i=1}^{C}\frac{TP_{i}}{TP_{i}+FN_{i}}, \end{array}$$

$$\begin{array}{c} \text{Macro-F1}=\frac{2\cdot \text{Macro-Prec}\cdot \text{Macro-Rec}}{\text{Macro-Prec}+\text{Macro-Rec}}. \end{array}$$

In the above metrics, *TP* denotes True Positives (samples correctly predicted as the positive class), while *TN* represents True Negatives (samples correctly identified as negative). The term *FP* indicates False Positives (negative samples erroneously classified as positive), and *FN* signifies False Negatives (positive samples mistakenly predicted as negative). For multi-class scenarios, *C* corresponds to the total number of classes, with $TP_{i}$, $FP_{i}$, and $FN_{i}$ specifically designating the true positives, false positives, and false negatives for class *i* respectively.

Considering the trade-off relationships among the multiple indicators proposed in this paper and their task-specific weights, we utilize a comprehensive weighted indicator to assess model performance and integrate all evaluation metrics into a single score via entropy-weighted TOPSIS. In this process, the four metrics are first normalized, and objective weights $w_{j}$ are determined using the entropy weight method–where metrics with greater variability (i.e., lower entropy) receive higher weights. The weighted normalized decision matrix is then used to define the positive ideal solution *A*^+^ (representing the best performance) and the negative ideal solution *A*^–^ (representing the worst performance). For each model *i*, the Euclidean distances to these ideal solutions are computed as:


$$\begin{array}{c} D_{i}^{+}=\sqrt{\sum_{j=1}^{n}w_{j}(x_{ij}-x_{j}^{+}{)}^{2}}, \end{array}$$

and


$$\begin{array}{c} D_{i}^{-}=\sqrt{\sum_{j=1}^{n}w_{j}(x_{ij}-x_{j}^{-}{)}^{2}}, \end{array}$$

where $x_{ij}$ is the normalized value of model *i* for metric *j*, $x_{j}^{+}$ is the best value (ideal) for metric *j*, and $x_{j}^{-}$ is the worst value (negative ideal) for metric *j*. The final composite TOPSIS score is then calculated as:


$$\begin{array}{c} C_{i}=\frac{D_{i}^{-}}{D_{i}^{+}+D_{i}^{-}},\;0\leq C_{i}\leq 1. \end{array}$$

A higher $C_{i}$ indicates that the model is closer to the ideal performance across all criteria. This composite score offers a balanced and integrated assessment of the overall model effectiveness, complementing the individual performance metrics.

### 4.3. Research on general sports image classification framework

This general sports image classification experiment focuses on classifying each input image into its corresponding sport category. Unlike the later soccer match event classification task, it evaluates whether the proposed NaViT model based on knowledge distillation (Distill-NaViT) can distinguish among different sports categories, including cricket, wrestling, tennis, badminton, soccer, swimming, and karate. Sports images often contain substantial intra-class variations caused by athlete posture changes, camera viewpoints, background clutter, occlusion, and resolution differences, making this task suitable for evaluating the robustness and generalization ability of the proposed method.

The dataset used for this evaluation is sourced from publicly available web resources and has been de-identified. It contains 8227 labeled images across seven sports categories: cricket, wrestling, tennis, badminton, soccer, swimming, and karate. Each image is assigned a unique ID and accurately annotated. The images capture sports scenes at various resolutions and from different angles. To provide an intuitive view of the dataset, Figure 5 displays representative samples of the collected sports images, illustrating their visual diversity and real-world complexity. Table 2 provides the detailed settings of the dataset. Considering that data sources may contain multiple frames from the same video, random splitting could cause frames from a single video to be unevenly distributed between training and test sets, leading to data leakage and distorted performance. To mitigate this, data cleaning was performed by removing highly similar duplicate images.

**Table 2:** Number of labeled images per sport category.

Category	Sport	Number of images	Range of Image resolution	Average resolution
1	Badminton	1394	228×117−3848×3848	590.1×391.2
2	Cricket	1556	140×105−4577×3265	661.0×427.2
3	Tennis	1445	115×123−4352×3264	622.1×412.8
4	Swimming	595	190×102−3744×3744	552.6×354.9
5	Soccer	1188	114×117−4649×3099	651.8×447.7
6	Wrestling	1471	135×158−4857×3046	651.0×417.9
7	Karate	578	127×156−4238×2385	541.8×371.0

**Fig 5:**
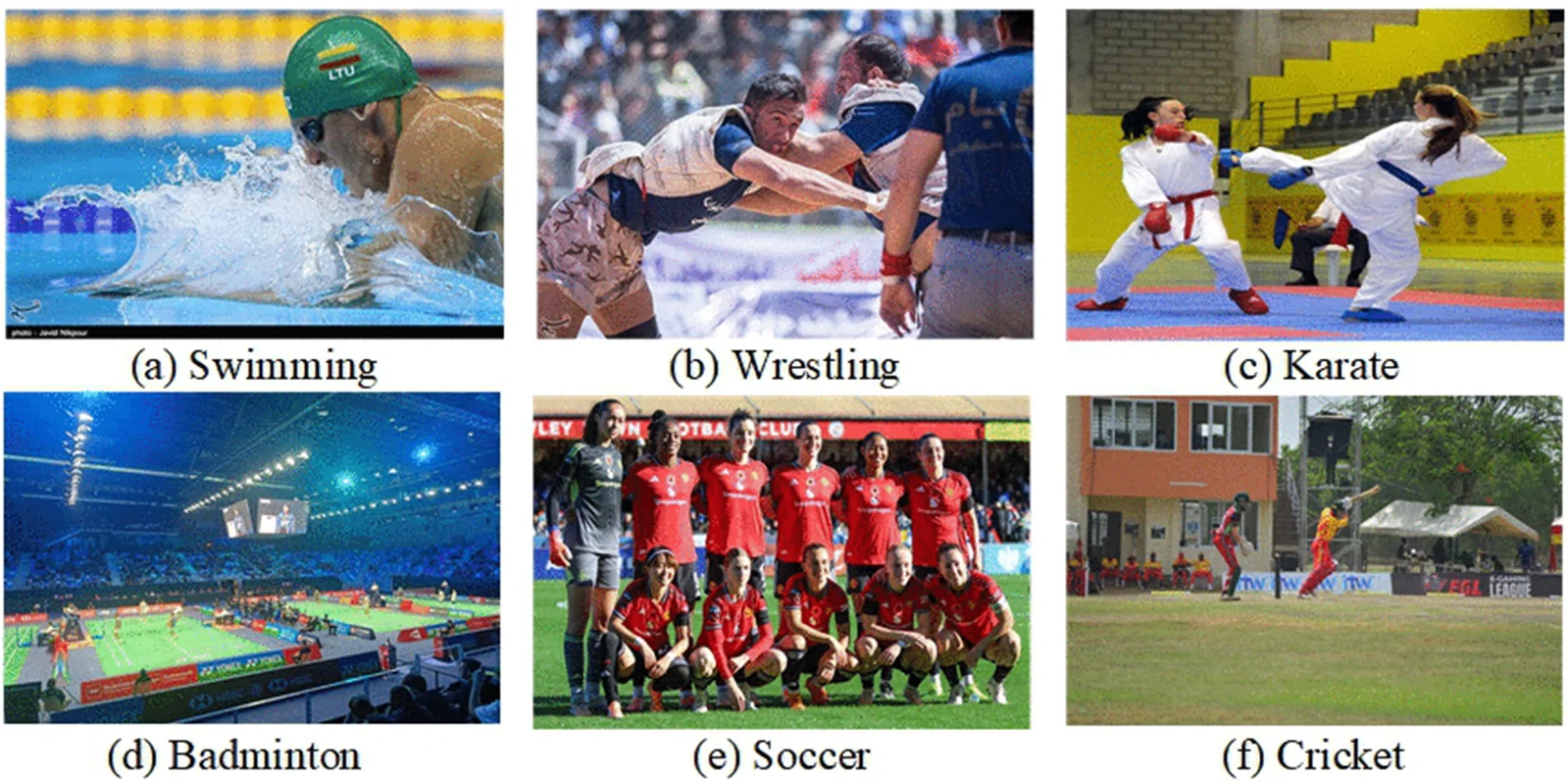
Representative samples from the sports image dataset. (Image credits for panels (a)–(d) are provided in Fig 1. The images in panels (e) and (f) are adapted from Wikimedia Commons under the Creative Commons Attribution 4.0 International (CC BY 4.0) License: (e) James Boyes; and (f) Amuzujoe. Full source and license information is provided in the Image Attributions section.).

#### 4.3.1. Comprehensive evaluation.

In all experiments, we split the dataset into training and testing sets at a 7:3 ratio through stratified random sampling, thereby maintaining a representative class distribution across both subsets. To validate the effectiveness of the proposed method against existing approaches, we conducted comparative experiments with the proposed Distill-NaViT model. For benchmarking, we selected several well-established models, including CNN-based architectures (ResNet-50 [26], MobileNet-V3 [27], and DenseNet-201 [28]) and ViT-based models (Swin-ViT [29], NaViT [19], and DeiT [30]).

These baselines were selected to enable a fair comparison among representative model families. The CNN baselines span three widely adopted convolutional design paradigms: ResNet-50 exemplifies residual learning and serves as a standard strong baseline for image classification; MobileNet-V3 exemplifies lightweight CNN architectures suited for resource-constrained deployment; and DenseNet-201 exemplifies dense feature reuse and powerful hierarchical feature extraction. Furthermore, DenseNet-201 also served as the teacher model in the proposed distillation framework, necessitating a comparison of its standalone classification performance with that of the student model.

The ViT-based baselines were chosen to ascertain whether the performance gain originates solely from the use of Transformer-based vision models or from the proposed architectural and training improvements. Swin-ViT represents hierarchical window-based self-attention, NaViT provides the closest native-resolution Transformer reference to the proposed architecture, and DeiT represents a data-efficient ViT framework closely related to knowledge distillation. Consequently, the selected baselines allow Distill-NaViT to be compared against both mature CNN architectures and closely related Transformer variants.

To ensure a fair evaluation under consistent conditions, all models were initialized with pretrained weights and underwent uniform transfer learning and fine-tuning. The training loss curve of the proposed model is presented in Fig 6, the iterative convergence curves for the first four evaluation metrics are presented in Fig 7, model performance across these metrics is illustrated in Table 3, and the TOPSIS evaluation results are provided in Table 4.

**Table 3:** Performance of the compared models on the general sports image classification task.

Model	Acc	Macro-Prec	Macro-Rec	Macro-F1
Distill-NaViT	0.9752	0.9754	0.9752	0.9753
NaViT	0.9560	0.9558	0.9587	0.9572
Swin-ViT	0.9243	0.9269	0.9243	0.9256
DeiT	0.9042	0.9047	0.9042	0.9044
DenseNet-201	0.8633	0.8648	0.8633	0.8640
ResNet-50	0.8805	0.8147	0.7618	0.7874
MobileNet-V3	0.4355	0.4181	0.4232	0.4206

**Table 4:** The comprehensive TOPSIS evaluation scores for the compared models.

Model	$D_{i}^{+}$	$D_{i}^{-}$	TOPSIS	Ranking
Distill-NaViT	0.0000	0.0471	1.0000	1
NaViT	0.0016	0.0456	0.9668	2
Swin-ViT	0.0043	0.0429	0.9094	3
DeiT	0.0061	0.0411	0.8715	4
DenseNet-201	0.0095	0.0376	0.7980	5
ResNet-50	0.0148	0.0329	0.6898	6
MobileNet-V3	0.0471	0.0000	0.0000	7

**Fig 6:**
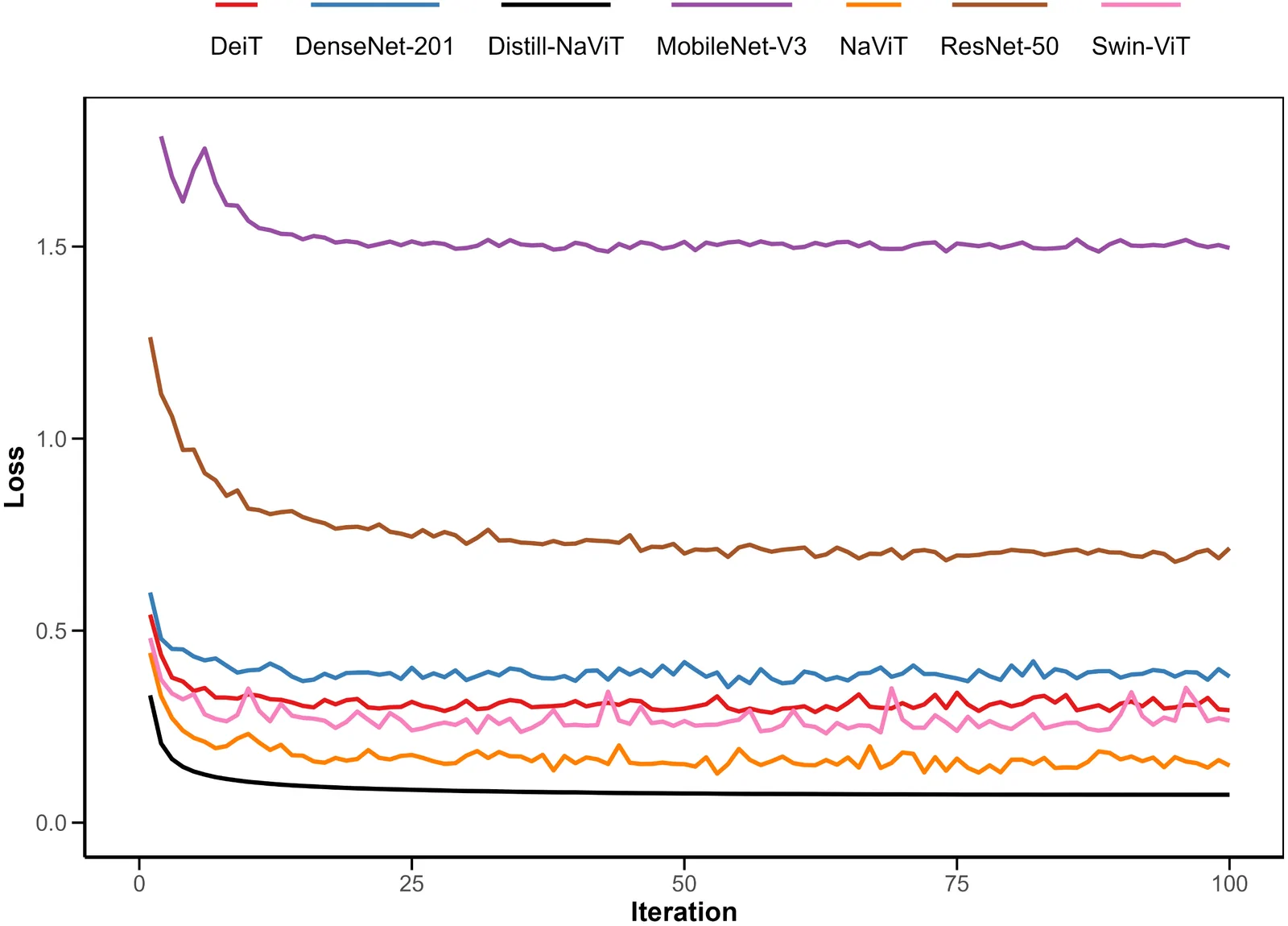
The training loss curves of the compared models.

**Fig 7:**
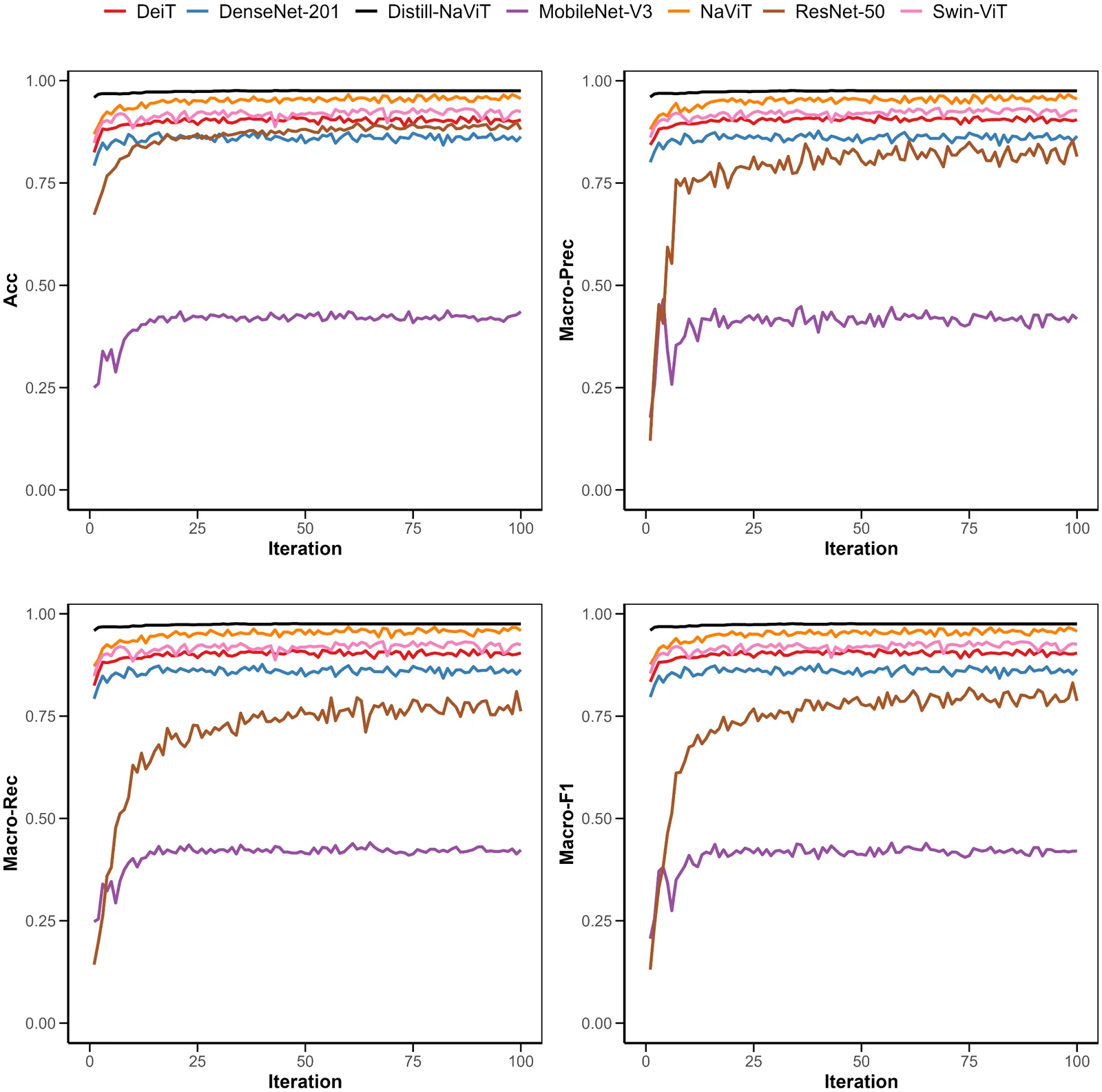
The iterative convergence curves of the first four evaluation metrics.

Fig 6 illustrates the training loss curves of all compared models. The proposed Distill-NaViT model demonstrates the fastest convergence and lowest loss throughout training, indicating superior optimization efficiency and generalization. This reflects the benefits of integrating transfer learning with knowledge distillation in improving convergence behavior and stability, especially in the context of complex sports image classification tasks.

Fig 7 and Table 3 illustrate the effectiveness of the proposed Distill-NaViT model. As shown in Fig 7, Distill-NaViT exhibits stable convergence on all four evaluation metrics, sustaining high values throughout training. Table 3 provides quantitative confirmation, with Distill-NaViT attaining the highest scores among all compared models: Accuracy (0.9752), Macro-Precision (0.9754), Macro-Recall (0.9752), and Macro-F1-score (0.9753). Distill-NaViT holds clear advantages over alternative architectures. It notably exceeds CNN-based models such as ResNet-50 in Accuracy and surpasses NaViT in Macro-F1-score. In comparison with MobileNet-V3, it achieves considerably lower classification error rates. These improvements arise from the integration of transfer learning and knowledge distillation, which strengthens feature extraction capabilities. The consistent performance on the test set indicates effective generalization across a range of sports image scenarios.

Table 4 summarizes the comprehensive evaluation scores using the TOPSIS method. Distill-NaViT achieves the highest score and ranks first, significantly ahead of other models. This composite ranking, based on entropy-weighted evaluation metrics, provides strong evidence for the robustness and superiority of the proposed model in real-world multi-category sports image classification scenarios.

#### 4.3.2. Ablation study.

To isolate the individual contributions of transfer learning and knowledge distillation, we conduct ablation experiments using four model variants: the complete Distill-NaViT, Distill-NaViT without transfer learning (w/o Transfer Learning), NaViT with transfer learning, and the Baseline NaViT. The ablation results in Tables 5 and 6 further elucidate the contribution of each component in the proposed framework. All enhanced variants exhibit clear improvements over the Baseline NaViT, indicating that both transfer learning and the distillation-oriented architecture contribute positively to sports image classification. The Baseline NaViT achieves an Accuracy of 0.8755 and a Macro-F1 of 0.8867. Adding transfer learning alone raises these values to 0.9062 and 0.9414, respectively, suggesting that pretrained representations help the model adapt more effectively to the sports image domain. This effect is especially pronounced in class-balanced recognition, as reflected by the substantial increase in Macro-Recall from 0.8846 to 0.9481.

**Table 5:** Ablation results across four classification metrics.

Model	Acc	Macro-Prec	Macro-Rec	Macro-F1
Distill-NaViT	0.9752	0.9754	0.9752	0.9753
Distill-NaViT w/o Transfer Learning	0.9380	0.9390	0.9452	0.9412
NaViT with Transfer Learning	0.9062	0.9390	0.9481	0.9414
NaViT	0.8755	0.9193	0.8846	0.8867

**Table 6:** Ablation results across TOPSIS evaluation metrics.

Model	$D_{i}^{+}$	$D_{i}^{-}$	TOPSIS	Ranking
Distill-NaViT	0.0000	0.0663	1.0000	1
Distill-NaViT w/o Transfer Learning	0.0140	0.0524	0.7886	2
NaViT with Transfer Learning	0.0254	0.0409	0.6168	3
NaViT	0.0663	0.0000	0.0000	4

The Distill-NaViT variant without transfer learning also substantially outperforms the Baseline NaViT, attaining an Accuracy of 0.9380 and a Macro-F1 of 0.9412. Since this variant omits transfer learning and pretrained weights while retaining the distillation-oriented model design, the improvement indicates that the proposed structural modifications and knowledge distillation strategy can strengthen representation learning even without pretrained initialization. Notably, this variant yields a higher Accuracy than NaViT with Transfer Learning, although the two models achieve very close Macro-F1 values. This contrast implies that transfer learning mainly enhances general feature initialization and class-level recall, whereas the distillation-oriented structure provides stronger overall classification stability.

The complete Distill-NaViT model, which integrates both components, achieves the best performance across all four classification metrics, with an Accuracy of 0.9752, Macro-Precision of 0.9754, Macro-Recall of 0.9752, and Macro-F1 of 0.9753. Relative to Baseline NaViT, the full model improves Accuracy by 9.97 percentage points and Macro-F1 by 8.86 percentage points. Compared with the distillation-only variant, the addition of transfer learning further improves Macro-F1 by 3.41 percentage points, confirming the benefit of pretrained initialization and domain adaptation. Compared with the transfer-learning-only variant, the addition of knowledge distillation improves Accuracy by 6.90 percentage points and Macro-F1 by 3.39 percentage points. These results demonstrate that transfer learning and knowledge distillation are not individually sufficient; their combination is necessary to achieve optimal performance.

The TOPSIS results reinforce these findings. Distill-NaViT attains the ideal TOPSIS score of 1.0000 and ranks first, followed by the distillation-only variant and the transfer-learning-only variant. Both partial variants rank above Baseline NaViT, confirming that each component is beneficial. More importantly, the full model achieves the smallest distance to the ideal solution and the largest distance from the negative ideal solution, underscoring that transfer learning and knowledge distillation are complementary rather than redundant. Overall, the ablation study validates the design motivation of Distill-NaViT: pretrained knowledge improves domain adaptation, while the distillation-oriented architecture further fortifies robust and discriminative feature learning for complex sports image classification.

To further examine the classification behavior of each variant, Figure 8 presents their confusion matrices on the general sports image dataset. The baseline NaViT exhibits noticeable off-diagonal activations across several categories, indicating misclassifications among visually similar sports. NaViT with transfer learning reduces these off-diagonal entries. The Distill-NaViT variant without transfer learning further sharpens the diagonal, although a few categories still display minor confusion. By contrast, the complete Distill-NaViT produces a confusion matrix that is predominantly diagonal, with minimal off-diagonal entries across all seven sports categories. These visual patterns are in line with the quantitative results: transfer learning contributes to better class-level recall, knowledge distillation supports more discriminative feature learning, and their combination results in classification that is both accurate and balanced across classes.

**Fig 8:**
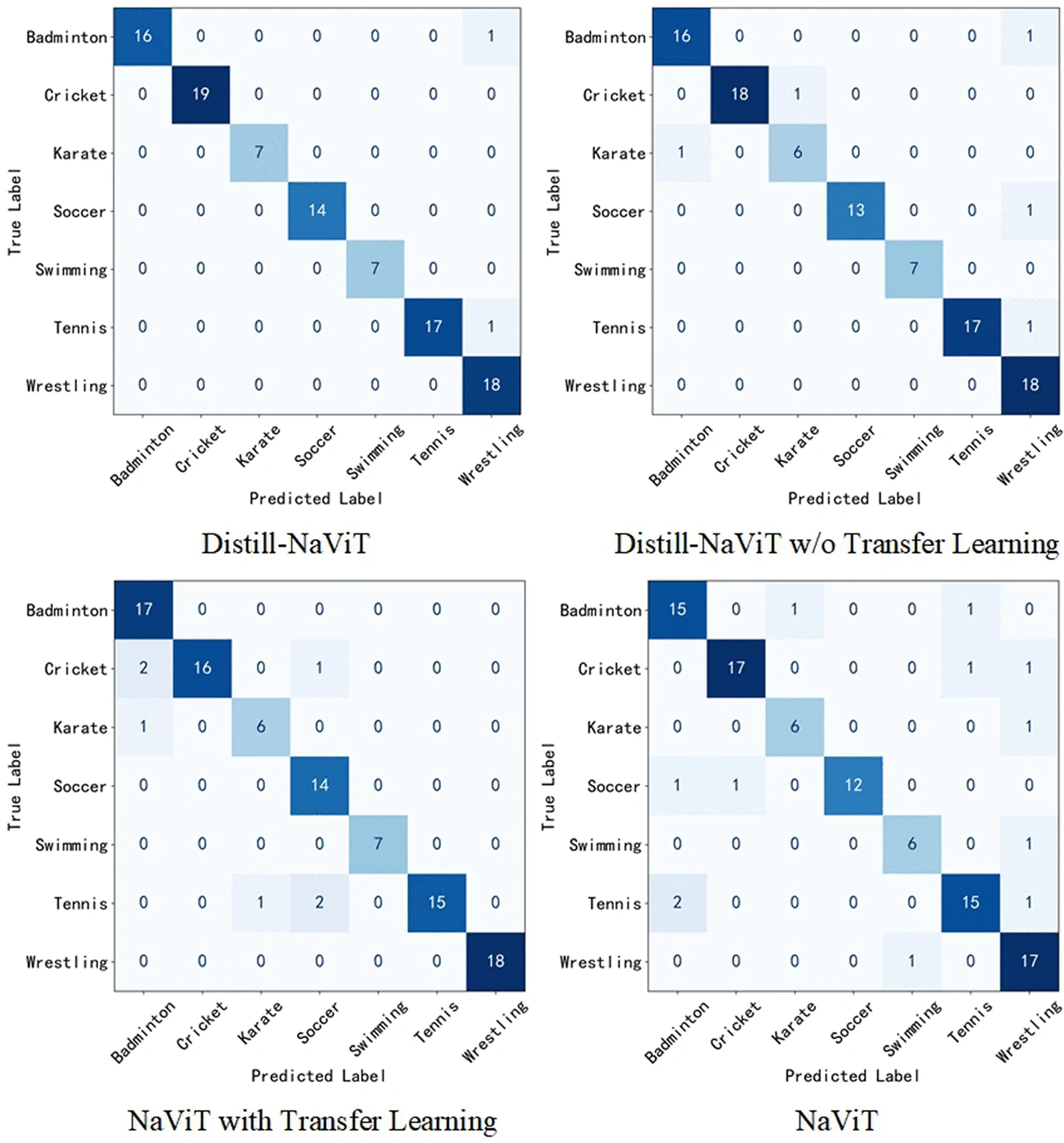
Confusion matrices of the ablation variants on the general sports image dataset.

### 4.4. Research on fine-grained event classification in soccer matches

We introduce a fine-grained soccer match event classification task to assess the proposed model in a more specific and demanding scenario, building upon its evaluation within the general sports image classification framework that emphasizes broad category-level recognition across various sports. Numerous practical sports vision applications demand not only recognizing that an image depicts soccer, but also identifying the specific match event, such as a goal, a red card, or a tackle. In contrast to broad sport-category classification, soccer match event classification entails higher inter-class visual similarity and subtler discriminative cues, as different events often share similar field backgrounds, camera viewpoints, player appearances, and match environments. Event discrimination may depend on localized posture variations, player interactions, ball positioning, or contextual cues. Accordingly, this task provides a complementary assessment of the model’s ability to capture fine-grained, sport-specific features and to extend from broad sport recognition to event-level understanding within a single sport.

The dataset used for this evaluation concentrates on distinguishing event types in soccer images. It comprises 9525 annotated images across three event categories: goals, red cards, and tackles. Each image is assigned an event label based on the primary match action depicted in the frame; all images have a resolution of 1280×720. To provide an intuitive view of the dataset, Fig 9 displays representative samples of the collected soccer event images, illustrating their visual similarity and the challenges of fine-grained event discrimination. In accordance with the data preparation procedure detailed in Section 4.3, data cleaning was performed prior to dataset splitting. Since soccer match images may contain multiple visually similar frames from the same video sequence, near-duplicate images were removed to reduce the risk of data leakage between the training and testing sets. Following cleaning, the dataset was partitioned into training and testing sets at a 7:3 ratio, using stratified sampling to preserve class distribution across subsets. The detailed composition of the soccer match event dataset is presented in Table 7.

**Table 7:** Description of the soccer match event dataset.

Event category	Number of images	Proportion	Resolution
Goal	8625	90.55%	1280×720
Red card	698	7.33%	1280×720
Tackle	202	2.12%	1280×720
Total	9525	100%	–

**Fig 9:**
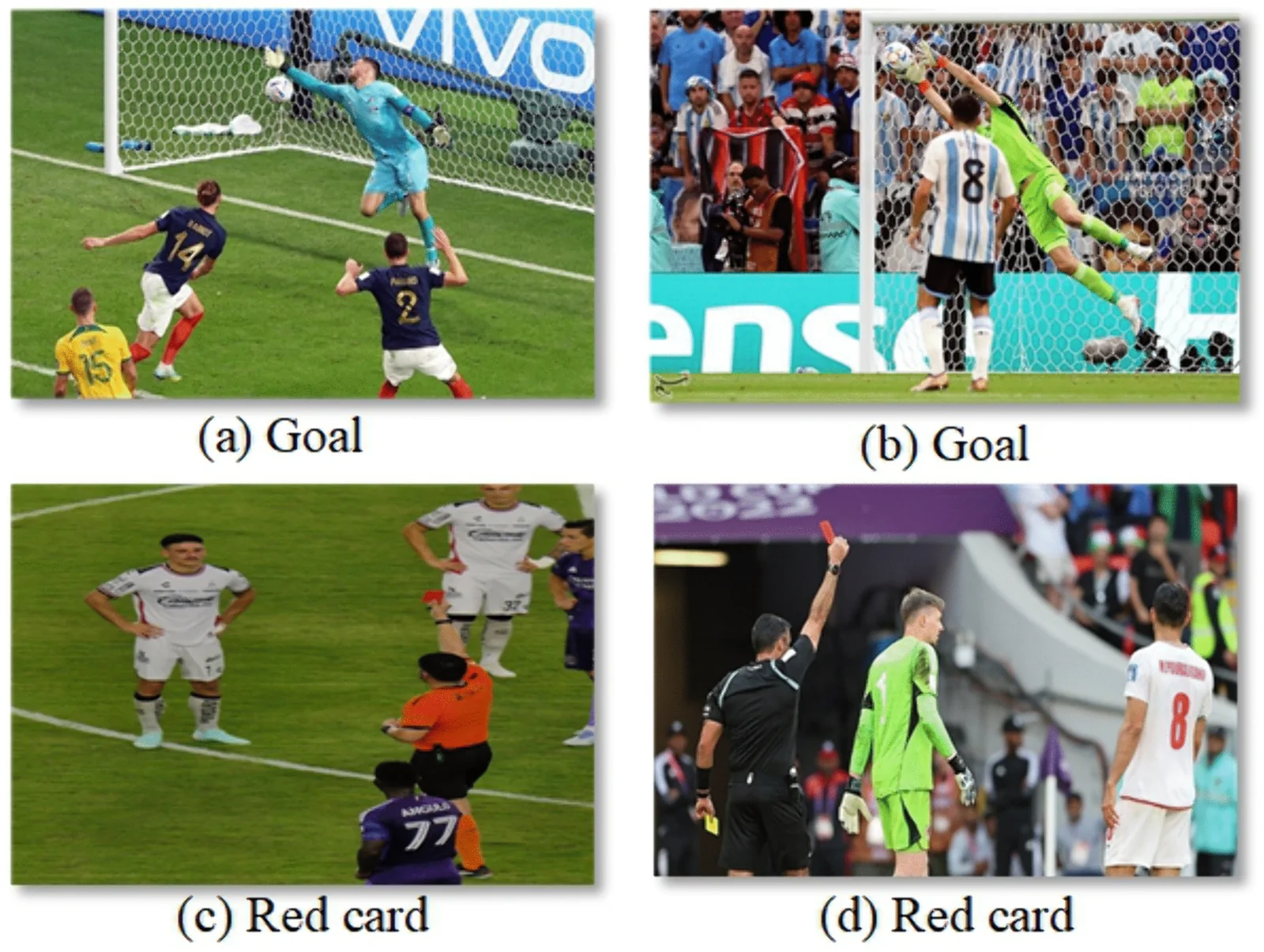
Representative samples from the soccer match event dataset. (The composite layout was created by the authors. The photographs in panels (a)–(d) were adapted from Wikimedia Commons under the Creative Commons Attribution 4.0 International (CC BY 4.0) License: (a) Hossein Zohrevand; (b) Hossein Zohrevand; (c) Keiteay; and (d) Hossein Zohrevand. The photographs were cropped/resized and arranged in the composite figure where applicable. Full source URLs and license information are provided in the Image Attributions section.).

#### 4.4.1. Multi-event classification performance.

A de-identified dataset of 9525 annotated soccer images from public web resources was employed, capturing diverse match scenarios with detailed event annotations. After data cleaning to prevent leakage, the dataset was partitioned into training and testing sets at a 7:3 ratio. We conducted systematic comparisons between our method and state-of-the-art models: CNN-based architectures (ResNet-50, MobileNet-V3, DenseNet-201) and Vision Transformer models (Swin-ViT, NaViT, DeiT).

Performance was evaluated using five metrics: Accuracy, Macro-Precision, Macro-Recall, Macro-F1-score, and the TOPSIS framework. Comprehensive results are presented in Tables 8–9.

**Table 8:** The model performance across the first four evaluation metrics.

Model	Acc	Macro-Prec	Macro-Rec	Macro-F1
Distill-NaViT	0.9863	0.9524	0.9921	0.9718
NaViT	0.9788	0.9365	0.9365	0.9365
Swin-ViT	0.9570	0.6515	0.6111	0.6307
DeiT	0.9454	0.6444	0.5556	0.5967
DenseNet-201	0.9299	0.6312	0.4444	0.5216
ResNet-50	0.9276	0.5865	0.5573	0.5715
MobileNet-V3	0.9026	0.2891	0.3333	0.3096

**Table 9:** The comparative performance of models across TOPSIS evaluation metrics.

Model	$D_{i}^{+}$	$D_{i}^{-}$	TOPSIS	Ranking
Distill-NaViT	0.0000	0.0842	1.0000	1
NaViT	0.0053	0.0793	0.9369	2
Swin-ViT	0.0444	0.0402	0.4750	3
DeiT	0.0493	0.0360	0.4223	4
DenseNet-201	0.0518	0.0327	0.3872	5
ResNet-50	0.0594	0.0285	0.3240	6
MobileNet-V3	0.0842	0.0000	0.0000	7

As Table 8 shows, the proposed Distill-NaViT model attains the highest performance across all four evaluation metrics, achieving an accuracy of 0.9863 and a Macro-F1-score of 0.9718. This advantage is especially evident in Macro-Recall (0.9921), indicating a strong capacity to identify positive event instances. NaViT ranks second across these metrics, while CNN-based models exhibit comparatively lower performance. Table 9 presents the TOPSIS analysis, in which Distill-NaViT obtains the highest TOPSIS score of 1 and the top ranking, with $D_{i}^{+}=0$ and $D_{i}^{-}=0.0842$ indicating close alignment with the ideal solution. These consistent results confirm the effectiveness of the proposed approach for soccer event classification.

Compared with the general sports image classification task in Section 4.3, the soccer event classification task poses a distinct challenge. In the general task, the model differentiates among seven sports with relatively clear inter-class differences, whereas the soccer event task requires discriminating three event types that share highly similar visual contexts. The proposed Distill-NaViT model delivers strong performance in both settings. The differing confusion patterns between the two tasks reflect the shift from broad category recognition to fine-grained event discrimination. The consistent advantage of Distill-NaViT across both tasks indicates that the combination of transfer learning and knowledge distillation facilitates both coarse-grained and fine-grained feature learning, which is beneficial for practical sports vision applications spanning multiple levels of recognition granularity.

#### 4.4.2. Critical event analysis: goal and red-card.

Fig 10 and Table 10 summarize the experimental results for goal and red-card event detection. Table 10 reports the model performance on four classification metrics, and Table 10 gives the TOPSIS evaluation for both event types.

**Table 10:** The model performance across TOPSIS evaluation metrics for goal and red-card event detection.

Soccer Match Event	Model	$D_{i}^{+}$	$D_{i}^{-}$	TOPSIS	Ranking
Goal Event	Distill-NaViT	0.0000	0.0522	1.0000	1
	NaViT	0.0093	0.0429	0.8226	3
	Swin-ViT	0.0093	0.0429	0.8226	4
	DeiT	0.0078	0.0449	0.8523	2
	DenseNet-201	0.0170	0.0361	0.6802	6
	ResNet-50	0.0125	0.0398	0.7614	5
	MobileNet-V3	0.0522	0.0000	0.0000	7
Red-Card Event	Distill-NaViT	0.0000	0.0512	1.0000	1
	NaViT	0.0060	0.0458	0.8846	2
	Swin-ViT	0.0108	0.0404	0.7885	3
	DeiT	0.0163	0.0355	0.6846	4
	DenseNet-201	0.0242	0.0359	0.5976	6
	ResNet-50	0.0193	0.0329	0.6307	5
	MobileNet-V3	0.0512	0.0000	0.0000	7

**Fig 10:**
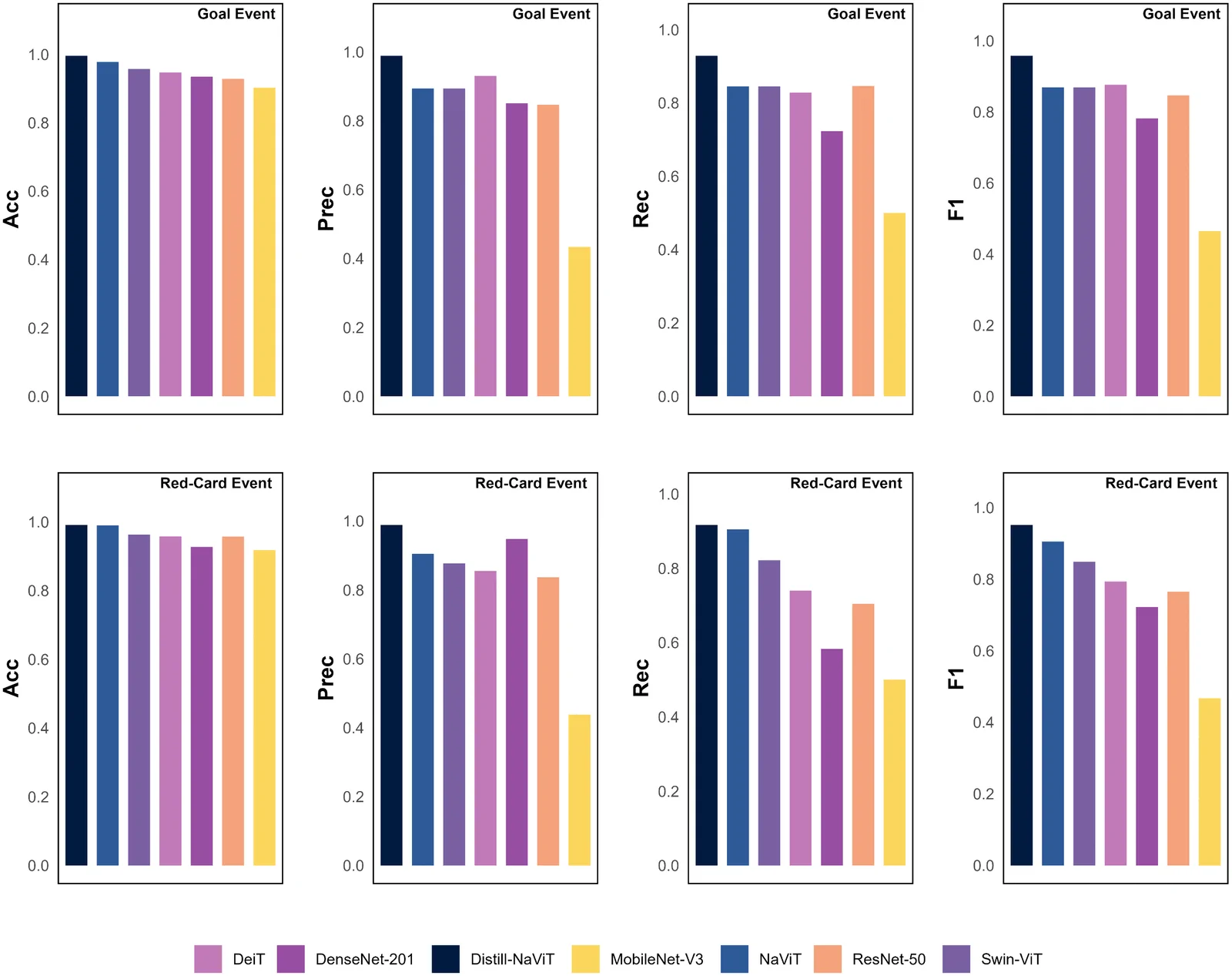
The model performance across the first four evaluation metrics for goal and red-card event detection.

Fig 10 compares model performance across four evaluation metrics for goal and red-card event detection. Distill-NaViT attains the highest scores across all metrics, whereas the remaining models exhibit varying outcomes between the two events. NaViT and DeiT deliver competitive yet event-sensitive performance, while CNN-based approaches display more pronounced disparities between precision and recall, indicating less balanced classification under fine-grained conditions. This asymmetry suggests that CNNs tend to concentrate on dominant visual patterns while overlooking the subtler cues required for the less represented event; in contrast, the self-attention mechanism in Transformer architectures enables a more balanced allocation of focus. Nonetheless, only Distill-NaViT sustains consistently high and well-balanced scores across both events, underscoring that the integration of transfer learning and knowledge distillation yields a more robust feature foundation and finer sensitivity to event-specific visual cues than either component alone.

Table 10 presents the TOPSIS evaluation results. Distill-NaViT consistently ranks first (TOPSIS = 1) for both goal and red-card detection. The rankings of the other models, however, shift between events. NaViT ranks second for red-card detection but drops to third for goal detection, while DeiT displays the reverse pattern, ranking second for goal detection and fourth for red-card detection. This reversal suggests that the two events pose distinct recognition challenges, and that NaViT and DeiT, although both are Transformer-based architectures, respond to these challenges in different ways. MobileNet-V3 consistently ranks last (TOPSIS = 0), reflecting its limited ability to capture the fine-grained features needed for event-level discrimination. The ranking shifts observed for other models across the two events indicate that goal and red-card detection depend on different visual cues, and that the combination of transfer learning and knowledge distillation in Distill-NaViT enables the model to capture both types of cues more stably than architectures that rely on a single learning paradigm.

## 5. Discussion

The experimental results indicate that the proposed Distill-NaViT framework achieves the strongest overall performance among the compared CNN- and ViT-based models on both the general sports image classification dataset and the soccer event classification dataset. The following discussion examines the mechanisms underlying these improvements and considers the limitations of the current framework.

The proposed parallel feedforward architecture separates target-task adaptation from distillation-guided representation transfer. Specifically, the trainable prediction block is optimized for the target classification task, whereas the independently parameterized distillation block remains frozen and provides a stable auxiliary representation. The attention-fusion mechanism employs the current prediction representation to compute input-dependent attention weights over the frozen distillation features and transfers the resulting weighted representation to the prediction pathway. Therefore, the contribution of the distillation representation varies according to the input while the frozen block itself remains unchanged. This mechanism provides an input-dependent means of regulating the contribution of the frozen distillation features to the prediction pathway.

Transfer learning and knowledge distillation serve complementary functions within the proposed framework. Initialization with ImageNet-pretrained weights provides transferable visual representations that facilitate adaptation to sports images. In addition, the transfer mapping aligns the student prediction logits with the teacher-logit space through the transfer loss during fine-tuning. In parallel, the fixed DenseNet-201 teacher generates temperature-softened class distributions, which are projected into a distillation token and incorporated into the student representation. Since these soft targets encode information about both target and non-target classes, they may provide supplementary supervisory information for distinguishing sports categories and soccer events with similar backgrounds, player appearances, or posture patterns. The ablation results support this interpretation: both partial configurations outperform the baseline NaViT, whereas the complete Distill-NaViT model achieves the strongest overall performance (Tables 5 and 6). These findings indicate that transfer learning and distillation-guided representation learning contribute complementary improvements rather than redundant effects.

Several methodological limitations should also be acknowledged when interpreting the results. First, the knowledge-distillation stage relies on a single fixed DenseNet-201 teacher, and the resulting supervision therefore depends on the quality and domain compatibility of the teacher’s output distribution. Teacher-specific errors may consequently be transferred to the student. In addition, projecting class-level teacher logits into a single distillation token may not fully preserve spatially localized or intermediate-layer information. Second, the transfer-learning stage relies on ImageNet-pretrained initialization. Although such initialization provides transferable visual representations, its effectiveness may be reduced when the source and target domains differ substantially. Third, the current experiments are based on individual static images and therefore do not explicitly model temporal dependencies, motion trajectories, or event evolution in sports videos. These limitations motivate further investigation of multi-teacher and feature-level distillation, adaptive loss balancing, more efficient multi-scale tokenization and attention mechanisms, and temporal or multimodal extensions.

## 6. Conclusion

This study proposes Distill-NaViT, a sports image classification framework that combines native-resolution visual representation, parallel feedforward processing, input-dependent attention-weighted feature fusion, transfer learning, and encoder-based knowledge distillation. The principal methodological contribution lies in integrating parallel feedforward processing and input-dependent attention-weighted feature fusion with transfer learning and encoder-based knowledge distillation, thereby combining pretrained visual representations with stable distillation guidance while retaining target-task adaptability. Experiments on the general sports image classification dataset and the soccer event classification dataset demonstrate that the proposed framework achieves strong overall performance relative to the compared CNN- and ViT-based models, while the ablation study further confirms the complementary contributions of transfer learning and the distillation-oriented architecture.

Despite these merits, the present framework is not without limitations, including dependence on the teacher model, coarse distillation granularity, constraints imposed by pretrained representations, and reliance on static images. Future work will investigate multi-teacher and feature-level knowledge distillation [31], adaptive optimization of the distillation and transfer objectives [32], and multi-scale attention and temporal modeling strategies [33]. It will further extend the framework to video and multimodal sports analysis. Collectively, the results suggest that integrating native-resolution Transformer representations with transfer learning and distillation-guided feature integration offers a promising approach for robust sports image classification under complex visual conditions.

### 6.1. AI tool usage disclosure

During the preparation and revision of this manuscript, the authors used OpenAI Codex using GPT-5.6 Sol in Ultra mode to assist with language polishing, organization of reviewer-response revisions, and improvement of textual clarity in selected parts of the manuscript, including the Abstract, Introduction, Methods, Experiments and Results, Discussion, and Conclusion sections. The AI tool was not used to generate original research data, conduct experiments, perform statistical analyses, create or alter primary results, or make scientific interpretations independently. All AI-assisted text was critically reviewed, edited, and verified by the authors, who take full responsibility for the accuracy, originality, cited sources, interpretation of results, and final content of the manuscript.

### 6.2. Image attributions

The composite layouts, annotations, labels, and other graphical elements in Figs 1, 5, and 9 were created by the authors. The photographic panels listed below were obtained from Wikimedia Commons and adapted for use in the composite figures. The source photographs remain copyrighted by their respective creators/rightsholders and are reused under the Creative Commons Attribution 4.0 International (CC BY 4.0) License. The photographs were cropped, resized, and/or arranged where applicable; no change in their substantive content was intended.

- Fig 1(a): Javid Nikpour / Tasnimnews. Source: https://commons.wikimedia.org/wiki/File:Swimming_at_the_2016_Summer_Olympics_%E2%80%93_Men%27s_200_metre_breaststroke_13.jpg
- Fig 1(b): Ali Dalkabadi. Source: https://commons.wikimedia.org/wiki/File:Ba-chukhe_wrestling_at_Zeynal-Khan_pitch,_Esfarayen,_Iran_20180404_Tasnim_01.jpg
- Fig 1(c): Wolfgang Burghardt, CSIT General Secretary. Source: https://commons.wikimedia.org/wiki/File:2019-07-04-Karate_Fighters_CSIT-World-Sports-Games.jpg
- Fig 1(d): Hoolen. Source: https://commons.wikimedia.org/wiki/File:Internationaux_de_France_de_badminton_2025_-_Huitieme_de_finales.jpg
- Fig 5(e): James Boyes. Source: https://commons.wikimedia.org/wiki/File:BHA_Women_2_MUFC_Women_3_02_11_2025-1007.jpg
- Fig 5(f): Amuzujoe. Source: https://commons.wikimedia.org/wiki/File:2023_African_Games_01_cricket_35.jpg
- Fig 9(a): Hossein Zohrevand / Tasnim News Agency. Source: https://commons.wikimedia.org/wiki/File:2022_FIFA_World_Cup_France_4%E2%80%931_Australia_-_(20).jpg
- Fig 9(b): Hossein Zohrevand / Tasnim News Agency. Source: https://commons.wikimedia.org/wiki/File:Argentina_vs_mexico_atajada_dibu.jpg
- Fig 9(c): Keiteay. Source: https://commons.wikimedia.org/wiki/File:Referee_showing_a_red_card.jpg
- Fig 9(d): Hossein Zohrevand / Tasnim News Agency. Source: https://commons.wikimedia.org/wiki/File:Iran_v_Wales_in_the_2022_FIFA_World_Cup_Match_17_-_16.jpg
